# Mapping menstrual health and hygiene progress in US schools: a systematic policy review and comparison across states

**DOI:** 10.3389/frph.2025.1589772

**Published:** 2025-08-20

**Authors:** April M. Ballard, Emily Wallace, Pranitha Kaza, Shannon Self-Brown, Matthew C. Freeman, Bethany A. Caruso

**Affiliations:** ^1^ Department of Population Health Sciences, Georgia State University School of Public Health, Atlanta, GA, United States; ^2^ Department of Health Policy and Behavioral Sciences, Georgia State University School of Public Health, Atlanta, GA, United States; ^3^ Department of Chemistry and Biochemistry, Georgia Institute of Technology, Atlanta, GA, United States; ^4^ Gangarosa Department of Environmental Health, Emory University Rollins School of Public Health, Atlanta, GA, United States; ^5^ Hubert Department of Global Health, Emory University Rollins School of Public Health, Atlanta, GA, United States

**Keywords:** period poverty, menstrual policy, adolescent health, menstrual equity, school-based health

## Abstract

**Introduction:**

Menstrual health and hygiene (MHH) policy initiatives have emerged as a key strategy to improve adolescent MHH, particularly through the expansion of state-level legislation aimed at increasing access to menstrual materials in K-12 schools in the United States (US). However, limited research has evaluated the implementation or effectiveness of these policies, and efforts to rigorously track and characterize existing policies remain limited. This study systematically reviewed and characterized state-level policies concerning menstrual material access in K-12 schools.

**Methods:**

We conducted a comprehensive search of all 50 US state government websites and legal databases to identify relevant legislation. Using MHH domains covered by the indicators recommended by the Global MHH Monitoring Group, we characterized policies. We also estimated policy reach by state and overall using National Center for Education Statistics enrollment data.

**Results:**

We found that 32 (64%) US states have enacted policies since 2017, which have the potential to improve MHH for approximately nine million, or 34%, of K-12 students. Most policies lack comprehensive coverage of essential MHH domains, including only three of the seven MHH domains on average.

**Discussion:**

These findings highlight the need for more rigorous research to evaluate the effectiveness of different policies and identify the best strategies for implementation.

## 1 Introduction

Globally, adolescents in both high- and low-income communities face many barriers to safe, hygienic, and dignified menstruation in school settings (1–6). Key challenges include no or inadequate access to menstrual material, a lack of private bathrooms, and insufficient menstrual health and hygiene (MHH) education (1, 7–10). These barriers negatively affect the health and well-being of menstruating students (1, 3, 11). Furthermore, when schools lack adequate MHH resources (e.g., menstrual material) and infrastructure (e.g., private bathrooms), they risk exacerbating existing health, economic, and social disparities by preventing adolescents from practicing necessary behaviors and having positive experiences during menstruation. Adolescents unable to effectively manage menstruation—particularly while in school—may experience declines in participation and attendance, which can reduce academic performance, increase grade repetition and dropout, and decrease economic potential and quality of life (1, 3, 7, 12–14). Since adolescence is a critical period for developing health capabilities (15–17), ensuring MHH needs are met at menarche and throughout puberty is vital for breaking cycles of inequity (11, 18).

Policy initiatives have emerged as a common strategy to address barriers to managing menstruation and to improve MHH among adolescents (4, 5, 19–21). Some countries have implemented comprehensive policies addressing multiple aspects of MHH, while others are narrower in scope (e.g., reducing or removing taxes on menstrual materials). However, many countries lack policies altogether (4, 5). The expansion of policy in the United States (US) has been particularly significant, overwhelmingly consisting of state-level legislation to increase adolescents’ access to menstrual materials in K-12 schools (21–23). Between 2017 and 2022, 21 states and territories passed policies focused on the provision of menstrual materials in schools (21). Amidst this rapid expansion of policies, there has been no systematic evaluation of these policies’ effectiveness or their implementation — aside from one study in Chicago Public Schools (24). Moreover, efforts to rigorously track and characterize existing policies remain limited.

This study addresses a critical gap in understanding MHH progress in the US by focusing on the most common adolescent-focused legislation: policies on menstrual material access in K-12 schools. This focus complements prior research on the only other type of adolescent-specific MHH legislation in the US, state school health education standards (25). Specifically, we systematically reviewed and characterized state-level policies concerning menstrual material access in K-12 schools using the seven domains covered by the indicators recommended by the Global MHH Monitoring Group (26) and building upon existing research and legislative tracking by non-profit organizations (e.g., Alliance for Period Supplies) and businesses (e.g., Aunt Flow). The seven domains were identified for integration into global and national monitoring efforts in response to the urgent need to understand unmet MHH needs among adolescents and to monitor progress across all aspects of MHH (26). In this study, *progress* refers to policy formulation and adoption. We do not assess the implementation, effectiveness, or impact of policies. Findings from this study will provide a benchmark for tracking progress and can help guide discussions on creating comprehensive and effective policies.

## 2 Assessment of policies

### 2.1 Search strategy

We systematically searched each US state's government website and three legal databases (Bill Track 50, LegiScan, and Casetext) for publicly available state legislation related to menstrual material access in K-12 schools (e.g., public and private elementary, middle, and high schools). Following Preferred Reporting Items for Systematic Reviews and Meta-Analyses (PRISMA) guidelines (27) (see Supplementary Table S1), our search targeted policies addressing menstrual material access because (1) they represent the most common adolescent-focused legislation in the US, and (2) a recent study reviewed the other primary type of adolescent-focused legislation, state school health education standards that may require MHH education (25). Generic search terms included [“menstrual hygiene”, “feminine hygiene”, “menstrual products”, “feminine products”, OR “period products”] AND [“schools”]. States with no relevant legislation were classified as having no policy. The search included all dates and concluded on August 12th, 2024.

### 2.2 Screening and selection of documents

To be eligible for inclusion in analyses, policy documents had to: (i) relate to the provision of menstrual materials in any K-12 schools, (ii) be from one of the 50 US states, (iii) be officially enacted by August 12th, 2024, and (iv) be the most recent and currently enacted version. We included three types of policies: appropriations (allocating or earmarking funds) (28), authorizations (permitting the use of funds) (28), and mandates (requiring specific actions, with or without funding) (29) related to MHH and K-12 schools (Table 1). Official state policy documents and accompanying fiscal notes were included in analyses. Policies that were solely focused on MHH education, were introduced with no resolution, had failed, were still in discussion, or had been amended or were no longer the current policy were excluded. States with failed or unresolved legislation were classified accordingly.

**Table 1:** Description of legislative terms and menstrual health and hygiene domains.

Term/domain	Description
Appropriation	A bill that allocates or earmarks funds to specific government departments, agencies, programs, or activities (Representatives)
Authorization	A bill that allows money to be spent by or on a specific government department, agency, program, or activity (Representatives)
Mandate	A bill that establishes an order or command that a specific government department, agency, program, or activity are required to comply, and may or may not include funds to adhere to the order or command ()
Menstrual materials	Materials to catch or absorb menstrual blood (26)
WASH facilities	Supportive sanitation facilities for caring for the body during menstruation, including having access to clean, private, and safe spaces to change menstrual materials (26)
Knowledge	Education about puberty and menstruation to equip adolescents with knowledge to help understand their bodies, to dispel fears around menstruation, and to support menstrual self-care (26)
Care for menstrual cycle-related discomforts and disorders	Resources to effectively manage menstrual pain (e.g., abdominal pain, cramping) and access to timely diagnoses, treatment, and care for menstrual cycle-related discomforts and disorders (26)
Supportive social environment	Environments that are free from stigma surrounding menstruation and provide access to individuals who can provide information, resources, or emotional support for menstruation (26)
MHH impacts	How menstruation affects adolescents’ day and class participation, or efforts to assess such effects (26)
Policy context	Existence of policies that include menstrual health and hygiene, and a budget with funds that are dispersed to schools in a timely and efficient manner (26)

WASH, water, sanitation, and hygiene; MHH, menstrual health and hygiene.

### 2.3 Data extraction

We developed a data extraction form in Excel using a mixed deductive and inductive approach that involved identifying a conceptual framework, piloting and refining the form, and ensuring consistency through independent extractions and reconciliation. First, we deductively identified critical components of effective and adequate MHH to assess policies, based on recommendations from the Global MHH Monitoring Group, a group of MHH experts and stakeholders who aim to develop indicators for and support countries in monitoring global progress in and out of school. The Global MHH Monitoring Group's recommendations—grounded in UNICEF's proposed operational pillars for MHH and definitions of “menstrual hygiene management,” “menstrual health and hygiene,” and “menstrual health”—identify five domains that are needed to achieve MHH: menstrual materials; water, sanitation, and hygiene (WASH) facilities; knowledge; care for menstrual cycle-related discomforts and disorders; and a supportive social environment. We also included two other domains—adolescent impacts of MHH and the policy context—based on the indicators recommended by the Global MHH Monitoring Group. Collectively, these seven domains provide a holistic framework for assessing adolescent MHH (26). Descriptions of the MHH domains are provided in Table 1.

Next, we employed an inductive approach by closely reviewing policies to identify additional themes. Three reviewers (EW, PK, and AMB) independently extracted data from the same five policy documents and compared their data to refine the extraction sheet and ensure consistency in extraction.

The finalized data extraction form captured policy details (e.g., year passed, type of policy), target schools and populations (e.g., public, grade levels), implementation cost estimates, funding provisions, and the seven MHH domains. Policies were independently reviewed by two researchers (EW and PK) using the pre-piloted data extraction form. Extraction inconsistencies were resolved by author AMB by re-checking the relevant documents and re-extracting the relevant data.

### 2.4 Data synthesis

Using R Studio v4.0.5, we generated descriptive statistics about policies in aggregate and sorted MHH domain data to characterize how policies addressed essential MHH requirements. We also estimated the potential reach of each policy using available National Center for Education Statistics [NCES; 2022–2023 for public schools (30), 2021–2022 for private schools (31)] data and the target schools outlined in policies. All data, as well as the pre-piloted data extraction form are publicly available (32).

## 3 Results

### 3.1 Overview of policies

**Figure 1:**
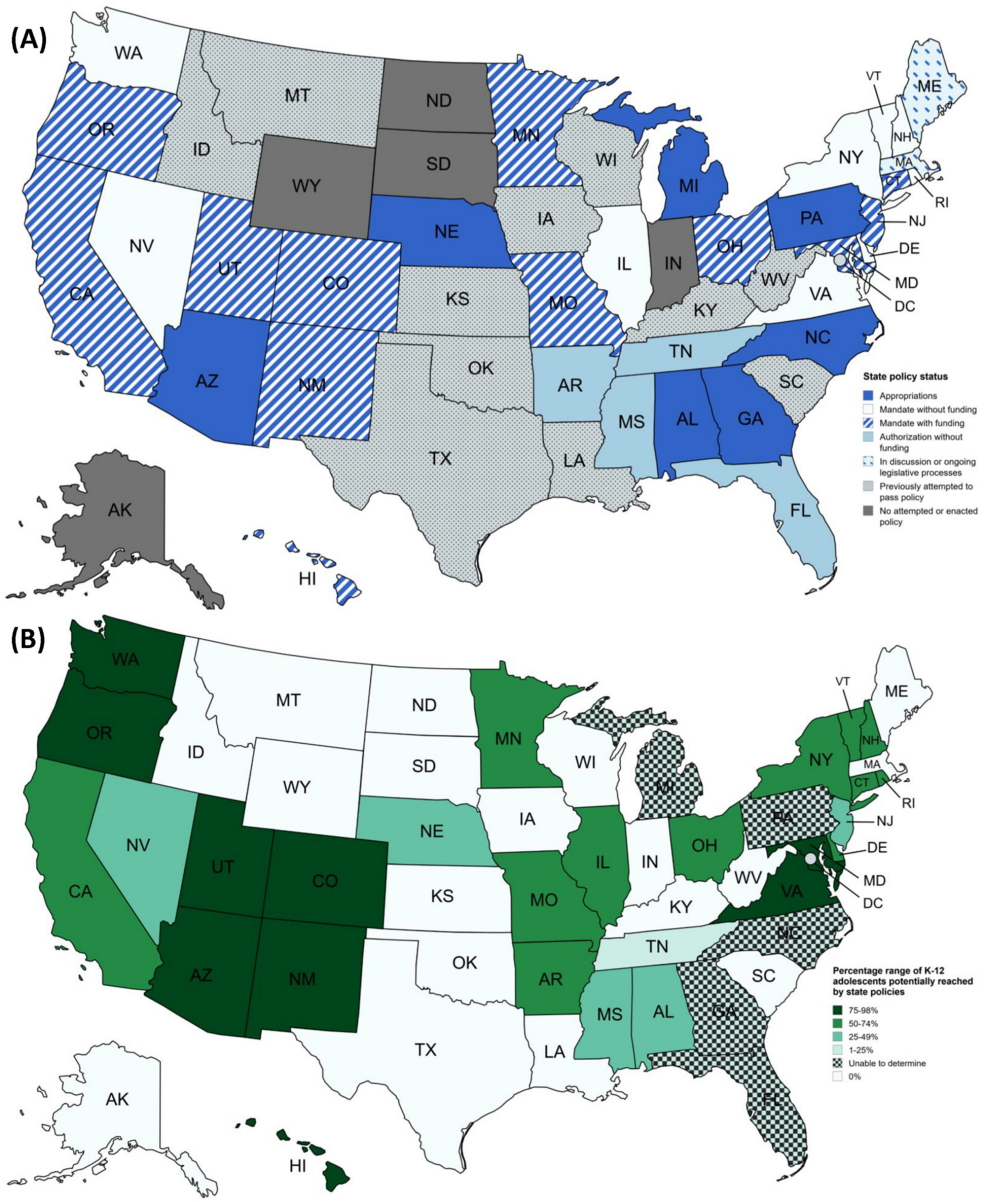
Maps of policy coverage in US states* **(A)** policy status as of August 12th, 2024, **(B)** estimated proportion of K-12 adolescents potentially reached by menstrual health and hygiene state policies. *Maps were created with mapchart.net.

**Figure 2:**
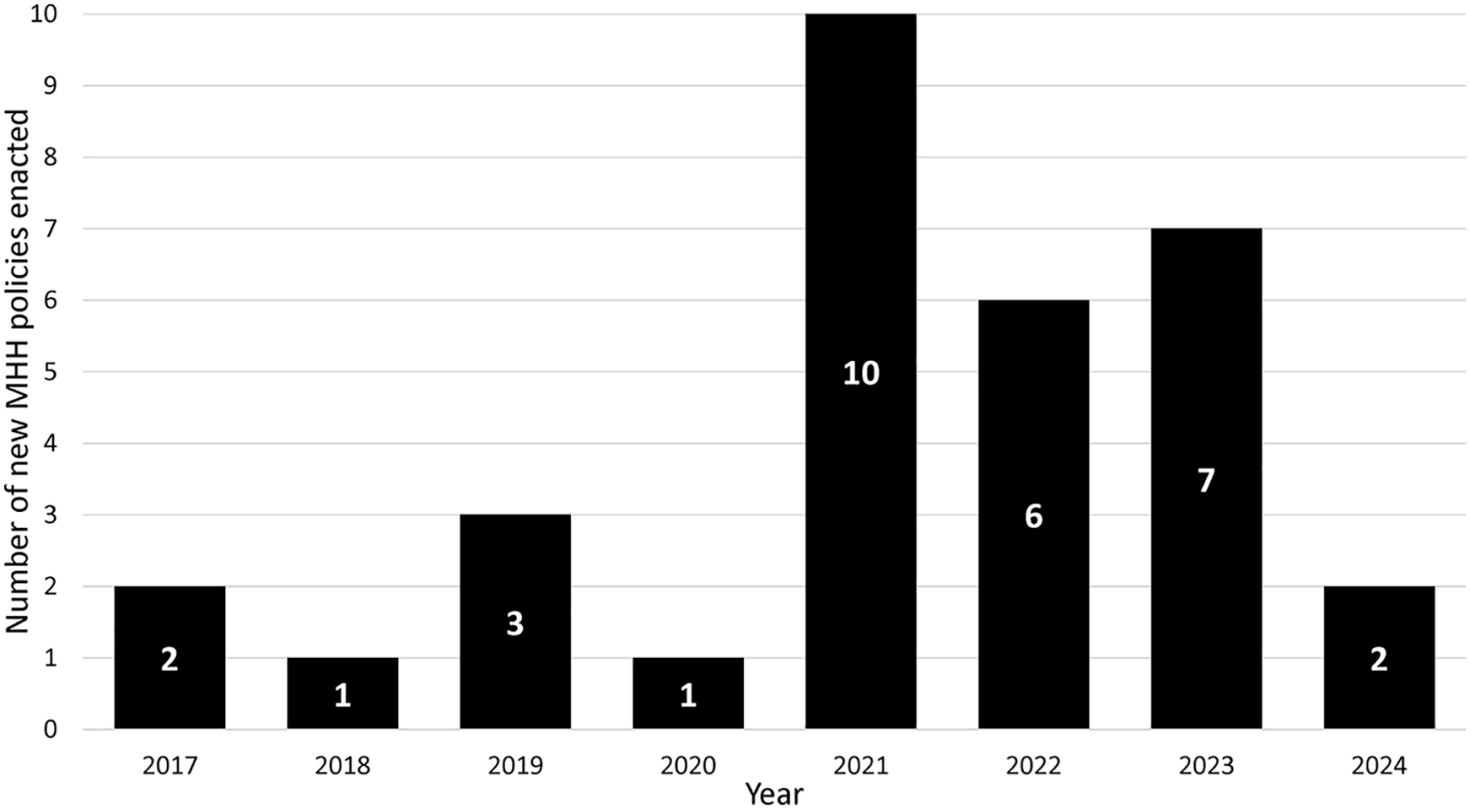
Number of menstrual health and hygiene (MHH) policies passed over time.

Our search revealed that 32 (64%) of 50 US states enacted policies to increase menstrual materials accessibility in schools (Figure 1A). These policies collectively have the potential to improve MHH for approximately nine million, or 34%, of K-12 students who can menstruate in the US (Figure 1B; Table 2). Additionally, 11 states (22%) unsuccessfully attempted to pass similar legislation and two (4%) had bills in discussion, meaning 45 (90%) US states had engaged MHH-related policy. Most policies were state mandates (21/32, 66%) requiring schools to provide menstrual materials, 12 of which (57%) included funding for implementation. Other policies were appropriations legislation (7/32, 22%) and unfunded authorizations (4/32, 13%). All active policies (*n* = 32) were enacted since 2017, reflecting a substantial increase in state level support for MHH in schools (Figure 2).

**Table 2:** Type of policy and select details by state based on the most recent and currently enacted version.

State	Type of policy^a^	Year enacted	Funding amount^b^	Potential percentage of K-12 students reached^c^	MHH domains that capture essential requirements for MHH						
					Materials	WASH	Knowledge	Discomforts or disorders	Supportive social environment	MHH impact	Funding
Alabama	AP	2022	$400,000	29	Yes	No	No	No	No	No	Yes
Arizona	AP	2023	$2,000,000	94	Yes	No	No	No	No	No	Yes
Arkansas	AT	2021	$0	52	Yes	No	No	No	Yes	No	No
California	FM	2017	NR	63	Yes	Yes	No	No	Yes	No	Yes
Colorado	FM	2021	$200,000	94	Yes	Yes	No	No	Yes	Yes	Yes
Connecticut	FM	2022	$2,000,000	71	Yes	Yes	No	No	Yes	No	Yes
Delaware	UM	2021	$0	58	Yes	Yes	No	No	Yes	No	No
Florida	AT	2023	$0	UA	Yes	No	No	No	Yes	No	No
Georgia	AP	2019	$1,500,000	UA	Yes	No	No	No	No	No	Yes
Hawaii	FM	2022	$2,000,000	82	Yes	No	No	No	No	No	Yes
Illinois	UM	2017	$0	64	Yes	No	No	No	No	No	No
Maryland	FM	2021	$500,000	87	Yes	Yes	No	No	No	No	Yes
Michigan	AP	2023	$1,000,000	UA	Yes	No	No	No	No	No	Yes
Minnesota	FM	2021	$2 per pupil	65	Yes	Yes	No	No	No	No	Yes
Mississippi	AT	2023	$0	49	Yes	No	No	No	No	No	No
Missouri	FM	2022	$1,000,000	50	Yes	No	No	No	No	No	Yes
Nebraska	AP	2024	$250,000	29	Yes	No	No	No	No	No	Yes
Nevada	UM	2021	$0	34	Yes	Yes	Yes	No	Yes	Yes	No
New Hampshire	UM	2019	$0	50	Yes	Yes	No	No	No	No	No
New Jersey	FM	2023	NR	49	Yes	Yes	No	No	No	Yes	Yes
New Mexico	FM	2023	$1,000,000	93	Yes	Yes	No	No	No	No	Yes
New York	UM	2018	$0	56	Yes	No	No	No	No	No	No
North Carolina	AP	2022	$250,000	UA	Yes	No	No	No	No	Yes	Yes
Ohio	FM	2023	$5,000,000	54	Yes	No	No	No	No	No	Yes
Oregon	FM	2021	$5,600,000	93	Yes	Yes	Yes	No	Yes	No	Yes
Pennsylvania	AP	2024	$3,000,000	UA	Yes	No	No	No	No	No	Yes
Rhode Island	UM	2021	$0	56	Yes	Yes	No	No	No	No	No
Tennessee	AT	2019	$0	9	Yes	Yes	No	No	No	No	No
Utah	FM	2022	$2,400,000	98	Yes	Yes	No	No	Yes	No	Yes
Vermont	UM	2021	$0	70	Yes	Yes	No	No	No	No	No
Virginia	UM	2020	$0	91	Yes	No	No	No	No	No	No
Washington	UM	2021	$0	77	Yes	Yes	No	No	No	No	No

^a^ Policy type: AP, appropriations; AT, authorization; FM, funded mandate; UM, unfunded mandate.

^b^ Funding amount: NR, not reported.

^c^ Potential percentage of K-12 students reached: UA, unable to estimate.

### 3.2 Delineation of roles and responsibilities

Schools and school districts were named as the main frontline implementers of policies in most states (30/32, 94%), with certain school staff (e.g., principals, nurses) being designated to determine where and how menstrual materials should be made available in schools in five policies (16%). Departments or Boards of Education were named as the policy administrators and/or enforcers in 15 (47%) states. Administrative responsibilities included establishing processes and parameters for schools and districts to apply for and receive funds to support implementation, allocating funds, reviewing applications to award funding, and reimbursing school purchases. Enforcement pertained to monitoring policy implementation and compliance and submitting reports to legislative entities.

### 3.3 Policy features by MHH domain

In aggregate, policies included administration and implementation approaches that covered six of seven MHH domains (Tables 2, 3), but on average only included three domains (range: 1–5; Table 2). While all policies (32/32) included information about menstrual materials, other domains were not as extensively covered. Approximately half included funding provisions (19/32, 59%) and WASH facilities (16/32, 50%). Actions for cultivating a supportive social environment (9/32, 28%), assessing MHH impacts (4/32, 13%), and delivering MHH education and training (2/32, 6%) were less common. No policy addressed reduction and care for menstrual cycle-related discomforts and disorders.

**Table 3:** Main policy features and descriptive statistics by MHH domains that capture essential requirements for MHH as recommended by the global MHH monitoring group (*n* = 32).

Domain	Main policy features	*N* states (%)
**Availability of menstrual materials in schools**		**32** (**100)**
	a. **Type of menstrual materials to be made available defined**	**28** (**88)**
	Included menstrual pads and tampons	28 (88)
	Included materials other than pads and tampons	9 (28)
	Included no cost or free menstrual materials	31 (97)
	b. **Approaches for making materials available specified**	**26** (**81)**
	Distribution in bathrooms	23 (72)
	Distribution in health centers via school nurses or other staff	6 (19)
	Distribution locations or individuals to be determined autonomously by schools or specific staff	5 (16)
	c. **Target schools and students specified**	**32** (**100)**
	Included public schools	32 (100)
	All K-12	7 (22)
	Subset of K-12*	25 (78)
	Included charter schools	21 (66)
	All K-12	5 (24)
	Subset of K-12^b^	16 (76)
	Included private schools	2 (6)
	All K-12	0 (0)
	Subset of K-12^c^	2 (100)
	Included specific students (i.e., females, students who can have a menstrual cycle, “at-risk pupils”)	4 (13)
**Access to supportive WASH facilities at school**		**16** (**50)**
	a. **Bathroom features discussed (e.g., availability, disposal options)**	**1** (**3)**
	b. **Availability of water and soap discussed**	**1** (**3)**
	c. **Types of bathrooms for menstrual material distribution specified**	**14** (**44)**
	d. **Percentage of number of bathrooms for menstrual material distribution specified**	**16** (**50)**
	General percentage or number of bathrooms with no specifics included	2 (6)
	100% of all bathrooms	1 (50)
	50% of all bathrooms	1 (50)
	Percentage or number of specific types of bathrooms	14 (44)
	100% of specific types of bathrooms	11 (79)
	50% of specific types of bathrooms	3 (21)
	1 or 2 specific types of bathrooms	4 (29)
**Delivery of MHH education and training in schools**		**2** (**6)**
	a. **Development of curriculum about menstrual materials mentioned**	**1** (**3)**
	b. **Provision of education discussed**	**1** (**3)**
**Reduction and care for menstrual cycle-related discomforts and disorders**		**0 (0)**
	**Not addressed by any policy**	**0** (**0)**
**Cultivation of a supportive social environment regarding menstruation**		**9** (**28)**
	a. **Menstrual material accessibility without stigmatizing students discussed**	**3** (**9)**
	b. **Provision of affirming and not-shamed-based MHH information mentioned**	**1** (**3)**
	c. **Ensuring student awareness about menstrual material availability included**	**6** (**19)**
**Assessment of MHH impacts**		**4 (13)**
	a. **Monitoring of the purchase and distribution of menstrual materials outlined**	**3** (**9)**
	b. **Evaluation of access to and quality and sufficiency of menstrual materials discussed**	**2** (**6)**
	c. **Evaluation of the impact of the provision of menstrual materials on student health and well-being discussed**	**1** (**3)**
**Allocation and disbursement of funds to support MHH policy**		**19** (**59)**
	a. **Provision of funding for schools to execute legislation included**	**19** (**59)**
	Recurring funding included	15 (47)
	One-time funding included	3 (9)
	b. **Types of MHH resources that funds can be used for specified**	**9** (**28)**
	Funds for menstrual materials	8 (25)
	Funds for dispensers	2 (6)
	Funds for disposal bins, WASH facilities, or other needs	0 (0)
	c. **Process for allocating and receiving funds detailed**	**9** (**28)**
	Established a grant program	4 (13)
	Established a reimbursement program	4 (13)
	Established a standard allocations formula	1 (3)

^a^ The subset of public schools includes: 4 with grades 3–12, 3 with grades 4–12, 3 with grades 5–12, 8 with grades 6–12, 1 with grades 9–12 at schools that are economically disadvantaged, and 4 with grades K-12 at schools that are economically disadvantages.

^b^ The subset of charter schools includes: 4 with grades 3–12, 3 with grades 4–12, 5 with grades 6–12, 1 with grades 9–12 at schools that are economically disadvantages, and 2 with grades K-12 at schools that are economically disadvantages.

^c^ The subset of private schools includes: 1 with grades 3–12 and 1 with grades 6–12.

#### 3.3.1 Availability of menstrual materials in schools

The type of menstrual materials, approaches for making materials available to students, and target schools and students were the main aspects of implementation elaborated on in policies (Table 3).

##### 3.3.1.1 Types of menstrual materials

Most policies (28/32, 88%) defined menstrual materials that can or must be made available, all of which included menstrual pads and tampons. Nine policies included flexible language (e.g., “but not limited to”) alongside specified materials to allow schools discretion in selecting materials. One policy (Ohio) also mentioned reuseable materials (e.g., cups, discs). Four policies did not define or specify the type of menstrual materials. No policies specifically noted types of materials that should not be included. All but one policy (Arizona) stated that materials should be provided at no cost.

##### 3.3.1.2 Approaches for making materials available

Most policies (23/32, 72%) primarily focused on implementation strategies to make menstrual materials available in school bathrooms at no cost. Specific distribution strategies within bathrooms largely were absent, although 10 policies mentioned the use or estimated cost of dispensers. Policies in Colorado, Michigan, Ohio, Virginia, and Washington schools, districts, school boards, or school principals to determine appropriate locations or designated individuals for distribution. Six policies targeted school nurses or other staff (e.g., counselors, teachers), either to serve as the sole access point in schools (Florida, Mississippi) or to complement bathroom distribution (Colorado, Vermont, Washington). Alabama's policy had conflicting details, stating that schools could be reimbursed for dispensers for distributing menstrual materials but that materials should be provided “to female students…through a female school counselor, female nurse, or female teacher.”

##### 3.3.1.3 Target schools and students

Every (32/32, 100%) state policy aimed to increase or authorize menstrual material provision in public schools, with most (25/32, 78%) targeting a subset of K-12 public schools (e.g., those serving grades 6–12). Only seven (23%) addressed the availability of menstrual materials in all state K-12 public schools. The remaining 25 pertained to a subset based on grade and/or poverty level. For example, New Hampshire's legislation required all public middle and high schools to make menstrual materials available in bathrooms; and Alabama's appropriations legislation established a Department of Education program to reimburse public schools with grades 5–12 who receive Title I funding to purchase and distribute menstrual materials. Tables 2, 3 provide additional details about schools targeted in policies.

Most state policies also included charter schools (21/32, 66%), five of which addressed menstrual material availability in all K-12 charter schools. Others mirrored public school specifications, pertaining to a subset of schools based on grade and/or poverty level. Only two policies (6%) included private schools. Both of which focused on specific grade levels: 6–12 in New York and 3–12 in Washington.

Four policies (13%) identified specific student populations to receive menstrual materials. Policies from Florida and Ohio mentioned that menstrual materials should be available to “female students”, and Delaware's mentioned students who can have a menstrual cycle. The policy from Michigan targeted “at-risk pupils,” which included those who faced challenges such as economic disadvantage and chronic absenteeism, among others. Michigan's policy further specified the number of materials students should receive: “at a minimum, 20 tampons or menstrual pads each month for the school year.”

#### 3.3.2 Access to supportive WASH facilities at school

Only two policies (6%; Oregon, Colorado) referred to specific bathroom features (e.g., availability, disposal options) and the availability of water and soap, even though the menstrual material availability in bathrooms was the primary implementation action discussed in policies. Oregon's policy defined the features of a bathroom: “Bathroom means a space with a toilet, a sink, and a trash receptacle that is privately accessible to students”. Colorado's policy mentioned that appropriated funds could be used to install and maintain disposal bins for menstrual materials.

The main WASH aspects in policies pertained to the number of bathrooms where materials should be made available. Fourteen policies (44%) specified that menstrual materials should be made available in specific types of bathrooms and approximately half of policies (16/32, 50%) specified the percentage or number of bathrooms where menstrual materials should be made available. Two policies included the percentage of bathrooms where menstrual materials should be made available generally: Minnesota's policy stated that materials should be in 100% of bathrooms and Delaware's stated 50% of all bathrooms. Most policies (14/16, 88%) included the percentage or number of specific types of bathrooms: 11 stated that materials should be in 100% of certain bathrooms, three stated that materials should be in 50% of certain bathrooms, and four stated materials should be in one or two specific bathrooms. Nine policies stated that materials should be available in 100% of bathrooms intended for all genders or that are gender neutral, with the remaining mentioning 50%.

#### 3.3.3 Delivery of MHH education and training in schools

The delivery of MHH education and training was only addressed in two policies (6%; Nevada, Oregon). Nevada's specifically required schools to develop a curriculum on menstrual material access. Oregon's policy required schools to provide “health and sexuality education that includes information on menstrual health,” and to provide and display menstrual product instructions within bathrooms. No details about specific knowledge or skills were included in the policy.

#### 3.3.4 Reduction and care for menstrual cycle-related discomforts and disorders

Menstrual cycle-related discomforts and disorders were not addressed in any policies.

#### 3.3.5 Cultivation of a supportive social environment regarding menstruation

Making menstrual materials available without stigmatizing students, providing affirming and not-shame-based MHH information, and informing students about the availability of menstrual materials were the main actions outlined in policies that related to cultivating a supportive social environment regarding menstruation. Policies from Connecticut, Nevada, and Oregon noted the need for materials to be accessible without stigmatizing those who request them. No specific actions were discussed, though Nevada's policy proposed that schools develop a plan to “ensure access and destigmatize the need for menstrual products.” Additionally, as discussed in Section 3.4, Oregon's policy required schools to provide positive and not-shame-based education and instructions on menstrual materials.

Six policies (19%; Arkansas, California, Colorado, Delaware, Florida, Utah) proposed strategies to ensure student awareness about menstrual material availability, which can help students feel comfortable requesting support. California's policy required schools to post a notice detailing policy requirements and contact information for an individual responsible for maintaining the supply of menstrual materials. Policies from Arkansas, Colorado, Delaware, and Florida focused on notifying students about the specific location of materials. Delaware's policy specifically required schools to publish and maintain menstrual material locations on school websites, whereas policies from Arkansas and Florida mentioned informing students generally. For example, Florida's policy states that “Participating schools shall ensure that students are provided appropriate notice as to the availability and location of the products”. Utah's policy was the least descriptive, stating that schools should inform students of the availability of menstrual materials.

#### 3.3.6 Assessment of MHH impacts

No policy included strategies to assess the impact menstruation had on students’ day or class participation. However, four (13%; Colorado, Nevada, New Jersey, North Carolina) outlined monitoring and/or evaluation strategies to be conducted by schools, district governing bodies, or state education departments. Monitoring strategies targeted specific materials purchased and distributed for reporting to state legislative bodies (e.g., The Senate and House of Representatives, Joint Legislative Education Oversight Committee). Evaluation strategies included assessment of menstrual material access, quality, and sufficiency, and the impact of the provision of materials on student health and well-being.

#### 3.3.7 Allocation and disbursement of funds to support MHH policy

Nineteen policies (59%) included appropriations or funding for implementation, most of which (15/19, 79%) were recurring funds. Twelve of the 21 (57%) enacted mandates established funding mechanisms for schools to execute legislative requirements. However, Colorado's policy only provided funding for certain schools to implement requirements. Unfunded mandates required schools to purchase materials using their existing budget or to obtain them through donations, gifts, grants, or partnerships.

Policies included $1.76 million for policy implementation on average (*n* = 16), though three policies (California, Minnesota, New Jersey) did not specifically state the amount of the funding to be allocated and funding varied widely (minimum: $200,000 [Colorado], maximum: $5,595,000 [Oregon). Eight of the 19 funded policies (42%) stated that funds were only for the purchase of menstrual materials. Two (Maryland, New Mexico) allocated some money to dispensers. The remaining policies did not include allocation details. No policies specifically allocated funding for disposal bins, WASH facilities, or other needed infrastructure, resources, or education.

Of the 19 policies that include funding, 47% detailed a process or stated that a process should be developed for funds to be allocated to or received by schools. Four policies established a grant program (Colorado, New Mexico, North Carolina, Pennsylvania). Colorado specified that schools with 50% or more students enrolled who are eligible for free or reduced-cost lunch and the Colorado School for the Deaf and the Blind must submit an application to the Department of Education that includes data concerning the number of students enrolled and the number of bathrooms on the property. Pennsylvania's policy outlined the same application process at Colorado, but public school entities with 25% or more students enrolled in free or reduced-cost lunch were eligible. New Mexico's policy stated that “grants of up to $5,000 will be awarded on a first-come, first-serve basis, prioritizing public school units that did not receive an award the previous fiscal year.” North Carolina's policy did not detail the grants program but required that the Department of Education establish and administer a grant program using existing resources and staff. Four other policies established a reimbursement program (Alabama, California, Maryland, Oregon) that required schools to file annual claims of costs. Minnesota uniquely included an allocations formula where schools received “$2 times the adjusted pupil units of the district for the school year” to purchase menstrual materials.

## 4 Discussion and actionable recommendations

Our systematic review of existing state legislation concerning menstrual material access in K-12 schools reveals progress in policy formulation and adoption, as well as the limitations of current MHH policies across the US. Thirty-two states have passed policies to increase adolescents’ access to menstrual materials in schools since 2017. However, the characterization of policies reveals that existing approaches do not comprehensively address all essential MHH domains as detailed by the Global MHH Monitoring Group. As a result, current policies may fall short in effectively supporting adolescent MHH in schools. Findings offer insights for improving MHH legislation, which can help to facilitate evidence-based policy development with the potential to significantly impact adolescent MHH. We offer five areas of consideration to improve existing policies and to guide the development of new policies: (1) establishing an MHH initiative and policy repository, (2) addressing all MHH domains comprehensively, (3) outlining clear actions and programmatic details, (4) including all relevant age groups and grade levels, and (5) providing adequate funding.

First, tracking and benchmarking MHH policies both for the US and globally is complicated by the lack of a centralized repository of initiatives and policy documents. Identifying policies during our review was challenging, with many only obtained after extensive searches. While this challenge is certainly not unique to MHH policies, the inability to find relevant policies complicates policy benchmarking and communication. Informal searches for resources related to best practices and lessons learned in MHH policy development and implementation also revealed gaps in information sharing. An open-access, full-text repository of initiatives, policies, guidance documents, and implementation toolkits for addressing adolescent MHH in schools would be a valuable step forward. The repository could include iterations over the years to enable assessment of changes made over time, as relevant, and could also support benchmarking for some of the Global MHH Monitoring Group's recommended indicators (26) and ideally connect to surveillance data to track progress in the coming years. We are ready to organize such a repository to address this gap and invite interested policy makers and researchers to contact us to contribute to and update our current database on OSF (32).

Second, many of the MHH domains are not addressed in the MHH policies included in this review, which is consistent with the evaluation of Illinois’ policy conducted in Chicago Public Schools (24), MHH policies in other countries (33, 34), and policies related to MHH education in the US (25). By comparing policy content to the essential MHH domains, we found that policies only covered three domains on average, with no single policy covering more than five of the seven domains. Additionally, a recent study on the only other type of adolescent-specific MHH legislation in the US—state school health education standards—found that the inclusion of MHH education in school health curricula is minimal and inadequate across states. Only three states cover menstrual materials (California, Michigan, and New Jersey) and three include menstruation management (Michigan, Oregon, Utah). Students in Oregon, for example, are taught about managing the physical and emotional changes that occur during puberty and about prioritizing personal care (25). A more holistic approach—one that extends beyond menstrual materials to include reducing and caring for menstrual cycle-related discomforts and disorders, providing education and training, fostering supportive social environments, ensuring menstrual-friendly WASH facilities, securing funding, and evaluating MHH impact—would significantly enhance the capacity of schools to effectively address the needs of menstruating adolescents. Aligning these efforts with ongoing advocacy to integrate MHH into school health education standards, which remains absent in most states (25), could further bolster the effectiveness of MHH policies.

Third, existing policies lack clear actions and programmatic details, limiting their practical implementation. Policies tended to be vague, often focused on the type of menstrual materials to be made available, how materials should be made available to students, and which schools and students will be targeted while omitting critical elements such as a detailed budget, implementation plan, evidence-based practices, or delegation of responsibilities. To improve the use of evidence-based practices and front-line priority setting, clear actions and programmatic details need to be outlined in policies. However, this will require research to determine what types of policies are effective and how to best implement those policies. While the limited number of effectiveness trials of menstrual material provision and educational interventions have demonstrated improved school attendance, MHH knowledge, and wellbeing, more rigorous research is needed to inform best practices for policy design and implementation (2, 4, 5, 35).

Fourth, current policies are not adequately responsive to the decreasing average age at menarche in the US (36), with few pertaining to all K-12 schools and only half including those younger than 11 years old. Research shows a significant trend toward earlier menarche over the past 50–100 years, with the prevalence of early menarche (before age 11) and very early menarche (before age 9) nearly doubling across birth years from 1950 to 2005. These trends are particularly pronounced among adolescents of low socioeconomic status and who are Black, Asian, or multi-racial (36). While only six policies explicitly target economically disadvantaged schools or students, these population-level trends underscore the importance of designing inclusive policies that consider both age and socioeconomic context. The inclusion of adolescents aged 9 and above in policies, with particular attention to low-income, Black, Asian, and multi-racial adolescents, would allow for timely intervention during critical developmental windows and would be responsive to widening MHH disparities.

Fifth, the absence of dedicated funding in nearly half of the policies reviewed, and the lack of budgetary provisions for disposal bins, WASH facilities, or other needed infrastructure, or education, represents a significant barrier to the sustainability and expansion of MHH programming. Without adequate funding to accompany policies, even well-designed policies are unlikely to achieve their intended outcomes. By including specific budget allocations for each MHH domain in policy provisions, state governments can better support the comprehensive MHH needs of adolescents and enable evaluation of policy impacts. Additional research is needed to assess the reach of current policies based on current funding, as well as to determine the optimal level of funding required to facilitate both effective implementation and long-term sustainability.

### 4.1 Strengths and limitations

This review used a mixed deductive and inductive approach to extract and characterize data, resulting in a comprehensive and rigorous synthesis of US state policies related to menstrual material access in K-12 schools. We intentionally used a structured yet flexible approach due to the novelty of the review and desire to capture in-depth information about policies. However, limiting our review to official state government policy documents focused on menstrual materials in K-12 schools presents some limitations. First, policies addressing other MHH domains that could complement those included in our review may have been excluded; though to our knowledge, other US MHH policies targeting adolescents are uncommon. Those that do exist are restricted to education about menstrual materials, menstrual management, and physiological aspects of menstruation (25). Second, the mere presence or absence of policies or strategies in a policy document does not necessarily reflect concrete action, or that the policy is achieving what it is intended to achieve. A well-recognized issue is the gap between what is articulated in official documents and what is actually implemented, and further, if the policy impacts the lives of those it is intended to serve. Additionally, MHH programs may be implemented in some states without a formal policy and these were not captured. Overall, the findings of this review indicate that few states have made significant steps in the development of a comprehensive set of strategies to address adolescent MHH in schools. However, in-depth evaluation of actual policy implementation, impacts, and resources allocated for state policies is needed to expand upon baseline data produced in this study.

## 5 Conclusions

Our findings indicate notable legislative expansion in the US toward supporting adolescent MHH, evidenced by 32 states enacting policies to support the provision of menstrual materials in K-12 schools. However, 18 states, representing approximately 7 million school age children who can menstruate, still lack any policy. Most policies lack comprehensive coverage of essential MHH domains, highlighting an urgent need for integrated, holistic approaches. Establishing an open-access, publicly accessible database of policy documents with regular systematic reviews of policy development could facilitate knowledge sharing and the development of more robust policies to strengthen adolescent MHH support.
